# The Academy of Stomatology

**Published:** 1895-02

**Authors:** 


### EDITORIAL.

### The Academy of Stomatology.

A new society with a new name, has recently been organized in Philadelphia, the objects of which, as stated in a circular issued a short time ago, is for the purpose of investigating and studying all matters connected with the mouth, its functions in health and their aberrations in disease. A prerequisite condition for the acceptance of all papers and communications read before it is that they shall be based upon original research, at least in some degree. The enforcement of this condition assures the selection of only papers possessing decided merit and which will add something to the total of our knowledge.

The academy already numbers among its members a majority of the most active and advanced practitioners of Philadelphia. They are men who have the best interests of dentistry at heart, and who energetically forward the work of dental professional progress to the best of their ability and means.

A commodious suite of rooms has been secured for the exclusive use of the academy. In connection with these a dental library and museum is being established together with a reading room where it is proposed to keep on file all of the leading dental periodicals of the world. The reading-room and museum will be open to members at all times during the day and evening. . . . The ultimate purpose of the academy is to establish a library and museum related to stomatology which will be consulted by dental practitioners throughout the entire country.

There has been for some time an idea entertained by quite a respectable number in the profession that a distinctive name, of broader significance than dentistry ought to be adopted. The word from which dentistry is derived simply means a tooth, and the words dentist and dentistry have about the same circumscribed meaning, while the word stoma, from which the word stomatology is derived, is mouth and all that immediately relates to it. This change, if adopted at all, will be slow. The word dentistry has been so long in use and so generally understood by every body that time will be required for the change.

In respect to euphony the word dentistry, perhaps, has a little the advantage but in most respects the new word, we think, is to be preferred.

